# Human Genetic Susceptibility to Invasive Aspergillosis

**DOI:** 10.1371/journal.ppat.1003434

**Published:** 2013-08-08

**Authors:** Cristina Cunha, Franco Aversa, Luigina Romani, Agostinho Carvalho

**Affiliations:** 1 Department of Experimental Medicine and Biochemical Sciences, University of Perugia, Perugia, Italy; 2 Department of Clinical and Experimental Medicine, University of Parma, Parma, Italy; Duke University Medical Center, United States of America

## Introduction

Aspergillosis includes a wide spectrum of diseases caused by fungi of the genus *Aspergillus* with clinical manifestations that range from colonization (e.g., aspergilloma), to allergic bronchopulmonary aspergillosis, to disseminated forms of infection. Invasive aspergillosis (IA) has been estimated to occur in 10% of acute myeloid leukemia patients during post-induction aplasia or consolidation therapy and after 5–15% of allogeneic hematopoietic stem cell transplants (HSCT) [1], [2]. Additional persons at risk for IA include recipients of solid organ transplants and patients with chronic granulomatous disease (CGD). Despite the significant progress attained in the management of this severe infection, its prevention, diagnosis, and therapy remain extremely difficult, rendering it a leading cause of death among immunocompromised patients. Additionally, concerns over antimold prescription and the remarkably high healthcare costs owing to its chronic course and mortality rates have been diverting clinicians from universal prophylaxis to risk stratification and preemptive approaches. This has inspired the search for novel individual prognostic factors, particularly genetic, to apply in the categorization of those most vulnerable to infection.

## Immune Recognition of *Aspergillus *: PAMPs, DAMPs, and Beyond

The physical barrier of the respiratory tract affords the first line of resistance against inhaled conidia of *Aspergillus*. In the event these escape the ciliated epithelium, conidia will then be challenged by cells of the innate immune system, including resident alveolar macrophages and dendritic cells (DCs), as well as recruited inflammatory cells, mainly polymorphonuclear neutrophils. These cells express a vast repertoire of pattern recognition receptors (PRRs) that sense pathogen-associated molecular patterns (PAMPs) and drive the secretion of proinflammatory cytokines and chemokines that arbitrate innate and adaptive immune responses. In the case of fungi, the cell wall is the main source of PAMPs owing to its dynamic composition and structural properties according to morphotype, growth stage, and environmental conditions [3]. Toll-like receptors (TLR)-2 (in cooperation with TLR1 and TLR6), TLR3, TLR4, and TLR9, and the C-type lectin receptors dendritic cell-specific intercellular adhesion molecule 3 grabbing nonintegrin (DC-SIGN), mannose receptor, and dectin-1 are among the PRRs recognizing fungal PAMPs including mannan, β-glucan, and nucleic acids [4]. Fungal sensing is further assisted by the action of collectins, ficolins, pentraxins, and complement components that act as opsonins and facilitate the interaction of phagocytes with fungi. Mammalian PRRs are also able to respond to products released from damaged host cells, including nucleic acids and alarmin proteins, collectively known as danger-associated molecular patterns (DAMPs). The unexpected convergence of PAMP- and DAMP-mediated molecular pathways raised the question of whether and how the host discriminates between them and the relative involvement of either one in inflammation, immune homeostasis, and mechanisms of repair during infection. Recent evidence has demonstrated that the immune system distinguishes fungus- and danger-induced immune responses, a mechanism relying on the spatiotemporal regulation of TLRs and the receptor for advanced glycation endproducts (RAGE) by the S100B alarmin [5].

Despite their undisputed relevance to pathogen resistance, innate immune mechanisms of pathogen detection may behave as double-edged swords, becoming detrimental as the result of hyperproduction of proinflammatory cytokines facilitating tissue damage or impairing protective immunity. This may explain why neutrophils, although indispensable in the implementation of the acute inflammatory response, may instead hamper resolution of infection through an excessive release of oxidants and proteases that may injure organs and promote fungal sepsis. Thus, although inflammation may serve to restrain infection, an overzealous reaction may contribute to pathogenicity and, paradoxically, favor disease progression. Along this line, genomic and transcriptomic approaches have revealed that fungal pathogenicity depends on mechanisms regulating fungal metabolism, morphogenesis, and response to stress in adaptation to the host environment. In particular, the ability of *Aspergillus* to adapt to hypoxic microenvironments has been found to involve modifications in fungal metabolism leading to the production of secondary metabolites that promote lung inflammation, exacerbate infection, and influence subsequent host immune responses [6].

## Genetic Variability of the Host and Susceptibility to IA

The inborn deficiency of the phagocyte nicotinamide adenine dinucleotide phosphate (NADPH) oxidase leading to CGD is the best known example of primary immunodeficiency with predisposition to IA [7]. As a result of the impaired production of reactive oxygen species, patients with CGD often develop IA, typically within the first decade of life. Of interest, these patients are uniquely susceptible to diseases with the *A. nidulans* complex, which are weakly virulent molds that rarely cause infection in immunocompromised patients. For most individuals however, genetic propensity to aspergillosis has a polygenic source. A polygenic variant by itself has a negligible effect on phenotype; only in combination with other remarkable predisposing variants (e.g., profound immunosuppression) do sizeable phenotypic effects arise.

In conformity with the crucial requirement of innate immunity for effective antifungal host defense, several studies have uncovered associations between genetic variants in components of the innate immune system and risk for IA [8]–[10] (Table 1). One classical example regards a donor haplotype in TLR4 reported to increase susceptibility to infection after HSCT, especially if combined with cytomegalovirus seropositivity [11]. Despite TLR4 polymorphisms having also been linked with chronic aspergillosis in immunocompetent individuals [12] and fungal colonization in HSCT recipients [13], their prognostic significance remains disputed. The fact that TLR4 ligand(s) in fungi are still unknown and the limited knowledge of the biological consequences of human TLR4 deficiency to antifungal immunity have been hampering the employment of TLR4 genotyping in risk stratification approaches. Most importantly, genetic variants affecting the function of innate receptors other than TLR4 have also been deemed relevant. Indeed, alongside the discovery of TLR3-mediated activation of protective memory CD8(+) T cell responses in experimental aspergillosis, a donor polymorphism impairing the expression of the human receptor was found to predispose to IA due to the inability of human DCs to efficiently prime memory CD8(+) responses to the fungus [14]. Additionally, and given the pivotal role of dectin-1 in fungal sensing, it is also not surprising that human dectin-1 deficiency has been reported to contribute to susceptibility to IA [15]–[17]. Interestingly, a stop codon polymorphism compromising the surface expression and dectin-1-mediated cytokine production displayed a cumulative effect toward risk for infection after HSCT when present concurrently in donors and recipients of stem cell grafts [16], a finding emphasizing the contribution of non-hematopoietic dectin-1 to antifungal immunity. As host damage perception is also fundamental for resolution of infection [5], genetic variants triggering hyperactive DAMP signaling, and presumably leading to uncontrolled inflammatory response to the fungus, were recently found to increase risk for IA [18]. Finally, and although positive associations between genetic variants in cytokine genes and vulnerability to IA have been reported [8], the lack of functional validation and the underpowered design of most studies precludes definite conclusions about the contribution of polymorphisms affecting cytokine production. One exception is the link proposed between a haplotype in CXCL10 and risk for IA in HSCT recipients [19]. Mechanistically, this haplotype was correlated with the inability of DCs to express CXCL10 and, interestingly, patients who survived IA displayed significantly higher CXCL10 levels compared to patients without disease.

**Table 1:** Genetic variants associated with increased risk for IA and probable pathogenetic mechanism(s) of susceptibility.

Gene(s)	SNP(s)	Amino acid change	Type of patients^1^	Cases (total patients)	Association [OR (95% CI), P value]	Probable mechanism(s)	Ref.
*AGER*	rs1800624	-	HSCT (D/R)	41 (223)	2.0 (1.0–3.8), P = 0.04 (D)2.0 (1.0–4.1), P = 0.05 (R)	Enhanced expression of RAGE	[18]
*CLEC7A*	rs16910526	Y238X	HSCT (D/R)Hematological	39 (205)21 (138)^2^	2.5 (1.0–6.5), P = 0.05 (D)3.9 (1.5–10.0), P = 0.005 (D+R)n.a., P = 0.02	Defective cell surface expression of dectin-1 and cytokine production	[15], [16]
*CLEC7A*	rs7309123	-	Hematological	57 (182)	5.5 (1.9–16.4), P = 0.001	Impaired expression of dectin-1 mRNA	[17]
*CXCL10*	rs1554013rs3921rs4257674	---	HSCT (D)	81 (139)	2.2 (1.2–3.8), P = 0.0072.6 (1.4–5.0), P = 0.0032.8 (1.6–5.2), P = 0.001	Impaired expression of CXCL10	[19]
*IL1A* *IL1B* *IL1RN*	rs1800587rs16944VNTR 86-bp(n)	---	Hematological^3^	59 (110)	15.4 (1.4–171.2), P = 0.02	Unknown	[25]
*IL10*	rs1800896rs1800871rs1800872	---	HSCT (R)^4^	9 (105)	9.3 (1.6–52.8), P = 0.01	Unknown	[26]
*IL10*	rs1800896	-	Hematological	59 (120)	4.5 (1.6–12.9), P = 0.001	Unknown	[27]
*MBL2*	‘MBL-low genotypes’^5^	-	HSCT (D)	15 (106)	7.3 (1.9–27.3), P = 0.003	Unknown	[22]
*MASP2*	rs72550870	D120G	HSCT (R)		6.4 (2.0–20.6), P = 0.002		
*PLG*	rs4252125	D472N	HSCT (R)	59 (194)	3.0 (1.5–6.1), P<0.0015.6 (1.9–16.5), P<0.001^6^	Unknown	[21]
*S100B*	rs9722	-	HSCT (D)	41 (223)	3.15 (1.61–6.15), P<0.001	Enhanced secretion of S100B	[18]
*TLR1* *TLR6*	rs5743611rs4833095rs5743810	R80TN248SS249P	HSCT (R)	22 (127)	1.2 (1.0–1.5), P = 0.041.2 (1.0–1.5), P = 0.021.3 (1.1–1.5), P<0.001^7^	Unknown	[28]
*TLR3*	rs3775296	-	HSCT (D)	42 (223)	2.4 (1.3–4.6), P = 0.007	Defective antigen presentation and activation of CD8(+) T-cell responses	[14]
*TLR4*	rs4986790rs4986791	D299GT399I	HSCT (D)^8^	33 (336)103 (366)	6.2 (2.0–19.3), P = 0.002 (discovery study)2.5 (1.2–5.4), P = 0.02(validation study)	Unknown	[11]
*TNFR1*	rs4149570	-	Hematological	77 (144)	n.a., P = 0.02	Impaired expression of TNFR1 mRNA	[29]
*TNFR2*	rs5745946	-	Hematological	54 (102)	2.5 (1.1–5.0), P = 0.03	Unknown	[30]

SNP – single nucleotide polymorphism; OR – odds ratio; HSCT – hematopoietic stem cell transplantation; D – donor; R – recipient; RAGE – receptor for advanced glycation end products; CXCL – chemokine (C-X-C motif) ligand; MBL – mannose-binding lectin; MASP – MBL-associated serine protease; PLG – plasminogen; TLR – toll-like receptor; TNFR – tumor necrosis factor receptor; n.a. – not available; VNTR – variable number of tandem repeats.

1 For patients that underwent HSCT, the source of the variant (donor, recipient, or both) associated with susceptibility to IA is indicated.

2 The controls for the association reported were not hematological patients, but healthy subjects of comparable Dutch ancestry.

3 Association with increased susceptibility to IA was observed for a haplotype comprising SNPs in the IL-1 gene cluster, namely *IL1A* rs1800587/*IL1B* rs16944/*IL1RN* VNTR 86-bp(n), but not for single loci.

4 Association with increased susceptibility to IA was observed for the absence of the ACC haplotype in rs1800896, rs1800871, and rs1800872, respectively.

5 ‘MBL-low genotypes’ correspond to a group of genotypes denoted by letters (O/O and LXA/O). LX represents an MBL promoter haplotype comprising SNPs of the MBL2 gene at positions −550 (H/L) and −221 (Y/X), known to influence transcription rates and to result in low concentrations of serum MBL. Nonsynonymous variants are collectively named O (including amino acid replacements at codons 52, 54, or 57) and cause a reduction of the MBL levels due to impaired assembly of MBL monomers into functional oligomers. A indicates the wild type.

6 Association results regard the comparisons DD *vs.* DN and DD *vs.* NN, respectively.

7 Association results regard R80T, N248S and S249P, and R80T or N248S and S249P, respectively.

8 Association with increased susceptibility to IA was observed for a *TLR4* haplotype (termed S4) that included both D299G and T399I.

Alternative strategies have been employed to uncover gene candidates for susceptibility to IA other than the “obvious” innate immune receptors and associated cytokines [20], [21]. For example, genetic mapping analysis of survival data of infected mice allowed the identification of plasminogen, a regulatory molecule with opsonic properties, as a fitting contestant for susceptibility [21]. Consequently, a nonsynonymous polymorphism in human plasminogen was found to increase risk for IA in HSCT recipients, particularly late after transplantation. Of interest, genetic and functional deficiency of other molecules with opsonic activity—e.g., mannose-binding lectin [22], [23] and PTX3 (our unpublished data)—have also been disclosed as major determinants of susceptibility to IA, pointing to a foremost contribution of humoral immunity in response to *Aspergillus*.

## Unveiling Human Susceptibility to IA: What Might the Future Hold?

Our existing knowledge of the genetic bases of susceptibility to IA derives from studies screening single variants in candidate genes using small patient cohorts. In addition, statistical issues with multiple comparisons and the lack of validation in larger, independent cohorts or via biological studies of disease mechanisms are further limitations of candidate gene association studies. As cutting-edge “omics” techniques are becoming affordable, multidisciplinary integrative approaches targeting variability in genome-wide association studies or expression in whole-transcriptomics studies may help to identify novel susceptibility signatures in otherwise unsuspected genes or pathways besides confirming those currently acknowledged. As “omics” have contributed to the identification of genetic susceptibility traits in cancer research, these techniques could be ultimately extrapolated with success to the field of invasive fungal diseases. Furthermore, only now are we beginning to fully grasp the significance of the microbiota and its interactions with the mammalian immune system in defining susceptibility to infection. Indeed, the structure and composition of lung microbial communities in patients at-risk was found to diverge significantly from that of healthy individuals [24], thus suggesting a likely susceptibility signature to IA that may involve a host–fungus–microbiota triad. All these state-of-the-art approaches however do not weaken the weight of functional validation. Given that “omics” studies by nature disregard all preceding knowledge about disease pathobiology, studies unveiling useful mechanistic insights into the relevant signatures found, be them genetic or biological, are still essential.

## Concluding Remarks

The identification of patient-specific prognostic signatures of susceptibility to IA in high-risk patients is currently one major priority in the fields of hematology and microbiology. Ultimately, the discovery of reliable markers of susceptibility consistently associated with risk for IA and functionally correlated with impaired antifungal mechanisms of the host may be a turning point toward innovative stratification strategies based on genetic screening or immune profiling to predict risk and severity of disease, efficacy of antifungal prophylaxis and therapy, and eventually contribute to the successful design of antifungal vaccines.
